# Using failure mode and effects analysis (FMEA) to generate an initial plan check checklist for improved safety in radiation treatment

**DOI:** 10.1002/acm2.12918

**Published:** 2020-06-25

**Authors:** Prema Rassiah, Fan‐Chi Frances Su, Y. Jessica Huang, Dan Spitznagel, Vikren Sarkar, Martin W. Szegedi, Hui Zhao, Adam B. Paxton, Geoff Nelson, Bill J. Salter

**Affiliations:** ^1^ Department of Radiation Oncology University of Utah Salt Lake City UT USA; ^2^ Avera Cancer Institute Sioux Falls SD USA

**Keywords:** FMEA, initial plan check, quality assurance

## Abstract

**Purpose:**

To apply failure mode and effect analysis (FMEA) to generate an effective and efficient initial physics plan checklist.

**Methods:**

A team of physicists, dosimetrists, and therapists was setup to reconstruct the workflow processes involved in the generation of a treatment plan beginning from simulation. The team then identified possible failure modes in each of the processes. For each failure mode, the severity (S), frequency of occurrence (O), and the probability of detection (D) was assigned a value and the risk priority number (RPN) was calculated. The values assigned were based on TG 100. Prior to assigning a value, the team discussed the values in the scoring system to minimize randomness in scoring. A local database of errors was used to help guide the scoring of frequency.

**Results:**

Twenty‐seven process steps and 50 possible failure modes were identified starting from simulation to the final approved plan ready for treatment at the machine. Any failure mode that scored an average RPN value of 20 or greater was deemed “eligible” to be placed on the second checklist. In addition, any failure mode with a severity score value of 4 or greater was also considered for inclusion in the checklist. As a by‐product of this procedure, safety improvement methods such as automation and standardization of certain processes (e.g., dose constraint checking, check tools), removal of manual transcription of treatment‐related information as well as staff education were implemented, although this was not the team's original objective. Prior to the implementation of the new FMEA‐based checklist, an in‐service for all the second checkers was organized to ensure further standardization of the process.

**Conclusion:**

The FMEA proved to be a valuable tool for identifying vulnerabilities in our workflow and processes in generating a treatment plan and subsequently a new, more effective initial plan checklist was created.

## 1 INTRODUCTION

Quality assurance (QA) is an integral and routine practice of radiation oncology departments. Over the years, QA has become more comprehensive to include not just equipment but also procedures, workflows, and communication.^1^, ^2^, ^3^

The initial plan check or second check done at the end of the treatment planning process is one such QA procedure implemented to ensure correct procedure and communication between various systems and teams (radiation oncologists, physicists, dosimetrists, therapists etc.) take place when a treatment plan is generated. Ford et al^4^ determined from studying the effectiveness of 15 different quality control (QC) tools that the initial plan check was the foremost effective tool in detecting potential error. The Medical Physics Practice Guideline (MPPG) for plan and chart review^5^ defines the purpose of the initial plan review as “to ensure compliance with prescription, no clinically significant deviations and that all information needed for the therapist to deliver the treatment is provided”. The American College of Radiology — American Association of Physicists in Medicine (ACR‐AAPM) technical standards for performance of radiation oncology physics for external beam^6^ defines the role of the qualified medical physicist in performing chart reviews and details essential items that need to be reviewed. These technical standards and guidelines provide recommendations on what needs to be checked, but are not department specific and may not be able to address vulnerabilities related to department‐specific workflows or procedures.

The use of checklists has been shown to reduce medical errors^7^, ^8^, ^9^ and has been advocated by the World Health organization (WHO). A practice guideline on the development, implementation, use, and maintenance of safety checklist was published by AAPM in 2015 in recognition of the importance of a well‐structured checklist.^10^ The purpose of a checklist in the initial plan review is to serve as an aid to ensure crucial steps in the process are not forgotten. However, the success of the checklist depends on it being relevant and effective enough that the users of the checklist do not succumb to checklist fatigue.^11^ Recently in our department, a treatment planning system (TPS) upgrade and introduction of supporting software have led to workflow and procedural changes. Several items had to be added to the initial plan checklist to address incidents that occurred and may occur due to these changes, leading to a significantly lengthened checklist. In order to avoid checklist fatigue while keeping the list’s effectiveness, we decided to review the checklist as a whole.

Failure mode and effects analysis (FMEA) is a widely used tool to analyze work processes methodically to identify vulnerabilities and their impact on safety.^4^, ^12^, ^13^, ^14^ Based on FMEA, resources can be concentrated on the most significant effect. In fact, the American Association of Physicists in Medicine’s (AAPM) Task Group (TG) 275^15^ recommends that each practice should assess local processes and identify key high‐risk failure modes.

We, therefore, decided to use FMEA to develop an initial plan checklist to ensure that effort is spent on checking high impact items, thereby making the process efficient and effective while avoiding checklist fatigue. To our knowledge, FMEA thus far has been used to review processes and workflows as a means to reduce risk^16^, ^17^, ^18^, ^19^, ^20^, ^21^ but has not been used for the purpose of creating a checklist.

Here we report our experience on using FMEA to create an initial plan checklist specific to our department while adhering to ACR requirements, MPPG guidelines, and taking into account TG275 recommendations.

## 2 MATERIALS AND METHODS

### 2.A Hardware and software systems

Two computed tomography (CT) simulators (GE lightspeed 16, GE Healthcare, Chicago, IL, USA and Somatom Definition, Siemens Healthcare, Erlangen, Germany) equipped with moveable lasers (LAP GmbH, Germany) are located within our department. Both scanners are used for simulation and with no specific patients directed to either scanners. Eclipse (version 15.5, Varian Medical Systems, Palo Alto, CA, USA) is used as both a virtual simulator and TPS. The algorithms used for dose calculation are Acuros version 15.5 for photon and electron Monte Carlo (EMC) version 15.5 for electrons. The TPS is complemented by a software package (MIM Vista, version 2.6, MIM, OH, USA), which is capable of performing functions such as image fusion, contouring, and dose summation. An Oncology Information System (OIS), (Mosaiq, version 2.6, Elekta, CA, USA) is used to transfer plan parameters required for treatment delivery to six linear accelerators: four Truebeams, one clinac iX (Varian Medical Systems, Palo Alto, CA, USA), and one Artiste (Siemens Healthcare, Erlangen, Germany). All linear accelerators are equipped with conebeam CT and the Artiste is equipped with CT on rails.

### 2.b Clinical team

In the recent 5 yr, our clinical team in radiation oncology has almost doubled. Currently, our department consists of 10 attending physicians, 9 radiation oncology residents, 11 physicists, 2 physics residents, 1 medical physicist assistant, 9 dosimetrists, 42 therapists, 7 nurses, and 3 medical assistants.

### 2.C Treatment planning process overview

**Fig. 1:**
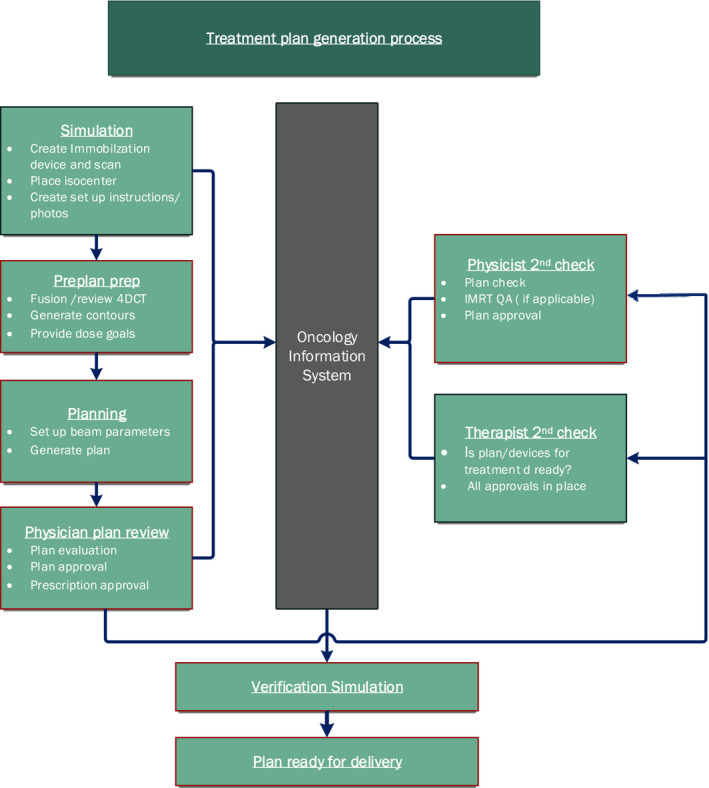
Treatment plan generation flow.

Immobilization devices and scanning technique (such as the use of contrast, 4DCT, etc.) are determined during simulation based on written simulation orders. The treatment isocenter is placed during simulation with the dosimetrist and radiation oncologist present. Setup instructions, which include photos, are created by the simulation therapist during simulation and placed in the OIS. Treatment plans are generated by the dosimetrist and reviewed in the TPS by the radiation oncologist. The dosimetrist then exports the treatment fields, reference CT, digitally reconstructed radiographs (DRRs), and a pdf of the plan to the OIS. The plan and prescription are then approved by the radiation oncologist in the OIS. All plans are second checked by a physicist prior to treatment who then signs off on the plan. Currently, there is no formal prereview prior to the second check in our workflow. The OIS is configured such that the system will prevent treatment if the prescription is not approved by the radiation oncologist or if the plan is not approved by both the radiation oncologist and physicist. A summary of the overall workflow is shown in Fig. 1.

### 2.D FMEA process

**Fig. 2:**
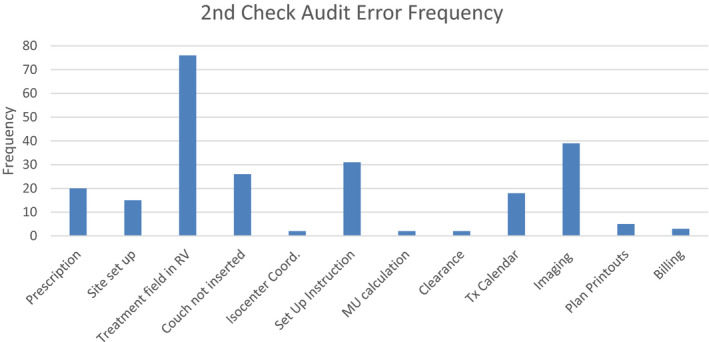
Frequency of recorded errors caught during the second check during 2017–2018. A total number of 210 errors were detected and an approximate of 5000 s checks were done during this 2‐yr period.

A team of physicists, dosimetrists, and therapists worked together to reconstruct the workflow processes involved in generation of a treatment plan beginning from simulation. The team then identified possible failure modes in each of these processes. For each failure mode, the severity (S), frequency of occurrence (O), and the probability of detection (D) were assigned a value and the risk priority number (RPN) was calculated as the product of these values. The values assigned were based on the report of AAPM task group 100 (TG 100).^22^ The range of scores for the S, O, and D was 1–10, leading to RPN values ranging from 1–1000. Prior to each team member assigning a value, the team discussed the scoring system as laid out by TG 100 to minimize the randomness in scoring. The consequence/s of each failure mode was discussed so that all team members understand the severity or repercussion of each failure mode. The resulting averages were also discussed as a group. A local database of errors that were identified and caught during the second check between 2017 and 2018, as shown in Fig. 2, was used to help guide the scoring of frequency. The team members were shown the error frequency so that they could use it as a guide when scoring. No formal correlation was made between the frequency score and the error frequency. There was a total of 210 errors detected and an approximate of 5000 s checks were done during this 2‐yr period. The scoring was done based on the relative frequency of these errors.

## 3 RESULTS

### 3.A FMEA and RPN determination

Twenty‐seven process steps and 50 possible failure modes were identified starting from simulation to the final approved plan which is ready for treatment. A summary of the identified failure modes and the associated RPN scores is shown in Table 1. Any failure mode that scored an average RPN value of 20 or greater was deemed ‘eligible’ to be placed on the second checklist. This cutoff value was chosen somewhat arbitrarily and was aimed at balancing between the workload and the ability to catch errors. In addition, any event with a severity score value of four or greater was also placed on the checklist. Based on TG 100, a severity score of fou would lead to a minor dosimetric error. Any value less than 4 means an error that does not have a dosimetric effect but may cause inconvenience to team members or patients. Prior to placing an item on the checklist, two things were considered: firstly, whether there is a simple workflow change that would change the likelihood of occurrence (decrease O); and secondly, if the failure cannot be lessened, whether software can automate the check to increase the detectability (decrease D) and remove the human element.

**Table 1:** Summary of identified process steps and failure mode. RPN scores or severity score that exceed the set value are in italics. Items that had a high score but was addressed via change of process or automatically checked are highlighted in gray.

Process	#	Process step	Failure mode	Average			
				RPN	S	O	D
Simulation	1	Make immobilization device	Immobilization device not sufficient for the accuracy of required for treatment.	18	3.3	2.8	2.3
	2	Scan patient	Did not scan the entire length of treatment.	9	3.0	2.5	1.2
			Wrong scan protocol used	18	2.0	2.0	3.8
			Wrong site	5	3.0	2.5	1.3
	3	Place isocenter	Did not place isocenter based on standard departmental protocol	14	3.3	1.2	1.5
	4	Mark isocenter on patient	Isocenter tattoo marked wrongly in the inf/sup direction, forgot (‐) sign	10	3.0	2.8	1.5
			Lasers are not in their zero positions when marking CT "0"	9	2.5	2.0	1.7
	5	Write setup instructions in Mosaiq	Wrong or incomplete setup instructions. That is, did not put in remove dentures, wrong shift to isocenter	*25*	4.0	2.7	1.3
Preplan preparation	6	Transfer CT to TPS	Incomplete dataset transferred	3	1.8	2.7	2.5
			Wrong dataset transferred ex 2nd scan, contrast,	*20*	3.4	2.7	1.5
	7	Place beams on isocenter (s)	Plan generated on wrong isocenter	8	3.0	2.7	1.8
Plan generation and review	8	Generate plan	Plan does not adhere to constraints	*66*	4.2	1.8	1.2
			Complicated plan generated, i.e., 14 fields when a simpler one is possible	10	1.8	2.8	2.8
			Previous RT tx dose not accounted for	*44*	6.4	2.8	1.2
			Couch not accounted for in plan	*25*	3.8	3.0	*6.4*
			Wrong prescription/ fractionation in plan (did not adhere to physician's order)	17	6.2	2.2	3.2
			Wrong algorithm, wrong CT to ED table, wrong grid size, wrong fusion, bolus not attached,	19	4.4	1.2	*5.6*
Export and preparation in OIS	9	Export plan pdf to Mosaiq	Wrong plan approved and exported to Mosaiq (multiple plans same patient)	*74*	6.4	3.6	1.8
			Wrong shift instruction (primary to bst)	13	3.6	1.4	2.0
			Plan incomplete (surface rendering, no DVH etc.)	15	2.2	1.4	3.0
	10	Export treatment fields to Mosaiq	Wrong field ID ( bolus/non bolus) as per dept. protocol	17	2.6	2.8	*4.4*
	11	Export DRR to Mosaiq	Isocenter placed wrongly on DRR	*31*	4.8	1.8	*6.4*
	12	Export DRR to Mosaiq	Suboptimal (image quality of) DRRs exported to Mosaiq	7	2.2	2.4	1.4
	13	Export ref CT to Mosaiq	Wrong reference CT exported	5	2.4	3.6	1.8
			No ref CT exported to Mosaiq	5	2.0	3.6	1.6
	14	Export reference to IGRT systems	Wrong isocenter exported to OIS, U/S , Exactrac, Calypso	17	5.0	2.3	3.5
	15	Export structure set to Mosaiq	Contours not exported as per physician ins.	14	3.0	2.4	1.4
	16	Import tx fields into Mosaiq	Incomplete import of treatment fields	6	4.2	2.0	1.0
			Wrong field ID (wrong number, missing B, etc)	17	2.8	2.4	1.0
			Wrong dose rate	13	1.6	2.8	1.8
			Wrong energy (energy changed in MQ)	7	5.2	2.6	2.4
			Wrong MU (mismatched MU on hand calculation)	12	4.2	1.4	1.2
			Wrong/missing FDA code for electron fields. Wrong wedge	11	2.6	3.8	1.4
			Imaging field parameter wrong or missing (wrong name, gantry angle, field opening size, SSD, etc.)	9	2.4	2.8	2.6
			Wrong/missing SSD	14	3.4	1.0	1.2
			Possible collision (lack clearance)	*26*	6.8	2.0	1.4
	18	Attached DRR to field	DRR not attached	8	2.2	2.8	1.2
			DRR attached wrongly	*22*	3.4	3.2	1.2
	19	Attach tolerance table	Wrong tolerance table attached	14	2.8	2.8	1.4
			Missing tolerance table	4	2.4	2.6	1.8
	20	Prep site setup in OIS	Missing site setup for electron fields	6	2.2	3.4	1.2
	21	Associate plan(s) with prescription(s)	Tx fields associated to wrong prescription	9	3.0	2.8	2.2
	22	Augment setup instructions if needed	Postsimulation isocenter shift is missing or isocenter added postsimulation	18	3.4	2.6	1.6
	23	Setup treatment calendar	Tx calendar setup wrongly (every other day tx/bolus, concurrent treatment, number of fractions, dose accumulation)	*28*	4.0	1.8	1.0
	24	Prescription approval	Prescription not complete (missing energy, technique, etc.)	9	2.6	2.4	1.0
	25	Imaging instruction	Sub optimal imaging instructions, i.e., frequency or imaging type not adequate	*27*	4.0	1.6	1.8
			Instruction for alignment not clear, overly complicated	10	3.0	2.2	2.0
			Inappropriate imaging instructions (Imaging modality not available in vault, etc)	10	2.4	3.0	2.0
	26	Special instruction	Missing info. Ex “need diode measurement”	18	3.4	2.2	1.6
Verification	27	IMRT QA/MUcheck	Failed IMRT QA	*25*	2	3.6	1.8

The severity scoring in our institution may differ from that of another institution depending on the work process. For example, the score of 3.0 may seem low for “wrong site scanned” but in our workflow, the simulation therapist scans the site to be planned based on written instructions by the physician given prior to scanning. The physician and the dosimetrist are present during simulation and confirm the site and isocenter location before the patient is taken of the table. Therefore, in our case the worst case scenario is that the patient is rescanned, resulting in a low severity score.

### 3.B Checklist creation

Initially, 18 items were deemed “eligible” to be placed on the checklist. One of these was “wrong MU calculation,” which is mainly caused by how the MUs are calculated for electrons. While the dose distribution is calculated using Eclipse’s implementation of EMC, the final MU setting is calculated using a hand calculation, a manual process. This was addressed by devoting physics resources into fully commissioning the EMC algorithm in Eclipse. The electron plan MU is now calculated by the TPS, eliminating a manual calculation and therefore the failure mode “wrong MU calculation” was removed from the checklist. Four other failure modes namely “dose constraints not being adhered to,” “treatment couch not accounted for,” “wrong calculation parameter,” and “clearance” can be checked by an automated software, Clearcheck (Radformation, NY, USA), and was placed under a single item in the checklist. The planner is required to run the automated software prior to the second check. The second checker will then review the report of the program as part of the check. Table 2 shows the new second checklist and the old checklist is shown in Table 3 for comparison.

**Table 2:** Second check Checklist as per the FMEA process.

Checklist category	#	Checklist item	What to check
Simulation	1	Setup instructions complete and correct	Complete, correct shift/table top, SSD, bolus thickness. Postsimulation isocenter shifts?
Plan generation	2	Planning dataset	Correct dataset used for planning (contrast/empty/full bladder).
	3	Previous treatment	If previously treated, is the previous treatment accounted for.
	4	Prescription	Is the prescription complete (Energy, technique and fractionation, dose)? Does prescription match the narrative summary and plan
	5	Clearcheck	Dose constraints adhered to, Couch inserted correctly, correct algorithm, grid size, clearance
Export and preparation in OIS	6	Correct plan exported to OIS	Was the plan that was approved by the physician in the planning system exported to the OIS?
	7	Export DRR to Mosaiq	Does the isocenter on the DRR look reasonable with plan?
	8	Export reference to IGRT systems	Is the isocenter exported to the imaging system correct (U/S, Exactrac, CBCT, Calypso etc.) and match plan?
	9	Treatment field	Do the treatment fields in OIS match plan
	10	Setup treatment calendar	Is the treatment calendar setup per prescription? Every other day tx/bolus, total fraction and dose
	11	Imaging instruction	Does imaging instruction seem reasonable (frequency/modality)?
Verification	12	IMRT QA/MU check	Did IMRT QA pass per dept. policy? Modulation OK? MU < 5X prescribed dose

**Table 3:** Shows the checklist used prior to the FMEA process.

Checklist category	#	Checklist item
Prescription	1	Prescription complete
	2	Prescription approved by AU
	3	Narrative summary match prescribed treatment
Plan	4	Tx plan matches prescription
	5	Hetero and bulk density correction
	6	Does the dose distribution look reasonable?
	7	Isocenter coordinates matches plan
	8	Does the DVH look reasonable
	9	Are the MUs reasonable as per prescription?
	10	Are the MUs checked?
	11	Clearance
OIS	12	Appropriate tolerance table used?
	13	Correct beam description and #
	14	DRRs correctly labelled
	15	Site setup approved
	16	Does MU match plan
	17	Tx parameter match plan
	18	Are set up instructions clear?
	19	Is the treatment calendar set up correctly?
	20	Wkly/Final/Protocol QCL available
	21	Special instructions clear
	22	Plan billing done
QA	23	Physics consult fulfilled?
	24	If IMRT, was the IMRT QA done prior to treatment

Significant additions to the second checklist generated by the FMEA process are item #3: previous treatment, item#8: iso “plan coordinates in IGRT systems” and item#11: “imaging instruction.” These are all items that were not checked with the previous checklist but the team deemed these items to be potentially unsafe for treatment if an error was made. Several items were consolidated in item #5: clearcheck. The items that were omitted that were previously checked in Table 3 are item #20: “weekly/final QCL” and #22: “plan billing.” Item “weekly/final QCL” is now automated, whereas “plan billing” was revealed to be checked by the billing department during the FMEA process, meaning it was a redundant check that could be dropped.

With the exception of item #22: “Plan billing done” and #23: “physics consult fulfilled” in the old checklist, all other items and more are checked in the new checklist. Despite this, the number of items in the new checklist is only 12 compared to the old checklist which has 24 items. This reduction in number was achieved by using clearcheck and consolidating several items that can be checked simultaneously into one item. The amount of time the second checker spends checking a plan with the new checklist may remain the same but the processes and the method of checking is more efficient than it used to be.

### 3.C Additional mitigation strategies

The process of systematically analyzing the treatment plan generation workflow and processes via FMEA resulted in the identification of other safety improvements, such as automation of certain processes, removal of manual transcription of treatment‐related information, and standardization of plan printouts in terms of content and organization. The purpose of standardizing the plan printout is to ensure that the required plan information is always available, should certain information be needed during treatment for patient setup, without the need for scrolling of the plan document back and forth in search of specific information. This standardization also facilitates a more efficient plan review by both physician and physicist. Prior to the implementation of the new checklist, an in‐service for all the second checkers was organized to ensure further standardization of the second check process. The “assessment’ module in Mosaiq is utilized to create a checklist with explanations on what needs to be checked. The second checker is required to complete the checklist by checking off each item electronically in Mosaiq.

## 4 DISCUSSION

Failure mode and effects analysis proved to be a very effective, systematic method of reviewing workflows and processes to identify vulnerabilities. Although not the initial objective of the team, workflow changes were implemented, certain checks were automated, and the standardization of certain processes was initiated.

Standardization plays an important role in error prevention.^23^ While the order and content of the plan printout has been standardized, the process of putting much of the report together is still manual. For example, the planner prints a PDF document of the treatment summary (for therapist time out), plan summary, dose distribution, dose constraints adherence etc. separately and then combines them in a given order before importing the report into Mosaiq. A script is currently being developed within Eclipse to generate the whole report with a single click which removes the manual step involved with report generation.

As shown in Fig. 2, errors made in the treatment field parameters of the OIS are one of the most frequently observed events. Considerable effort has been put into creating a more seamless export (from Eclipse) and import (into Mosaiq), where the planner does not have to interact with the treatment field once it is imported into the OIS. For example, block codes were setup in Eclipse, dose rates for certain treatments were defaulted in Eclipse, and initial treatment couch position (vertical, longitudinal and lateral) was setup as a default within Mosaiq so that the planner does not have to manually enter these values.

Clearcheck is a software module integrated within the Eclipse environment that is capable of automatically verifying several things. Although some of these items are not individually listed in the checklist, they are verified in Clearcheck and the report is attached to the plan in the OIS system for the second checker to review.

The utilization of third party software (Clearcheck), standardization of parameter transferred between Eclipse and Mosaiq, and treatment printouts are all efforts to transition from a somewhat manual workflow to an automated workflow. Automation has been shown to be one of the most effective ways of preventing errors in the radiation planning and treatment workflow.^24^, ^25^, ^26^, ^27^

The other by‐product of this process is the improved communication among the physicists, dosimetrists, and therapists and better understanding of each other’s work processes.

While there are many similarities between the second checklist generated in this study and the example second checklist given in TG275, there are items that are on our list which we think are specific to our department. For example, item #6 on our checklist “Was the plan that was approved by the physician in the planning system exported to the OIS?” In our workflow, the radiation oncologist chooses a plan in the TPS (if more than one plan is presented to the physician). The radiation oncologist then labels the plan that he or she likes with his or her initials. The dosimetrist then exports the plan with the label. There is a possibility for the wrong plan to be exported to the OIS. This may not be the case for a department where the TPS is an integral part of the OIS. There are also very explicit items in our checklist to ensure physician intent and plan is translated to the OIS which are not listed in TG275 (items # 7, #9, and #10).

The TG275 second checklist explicitly spells out every item in detail, whereas our checklist consolidates several checks in a single item. For example, TG275 spells out prescription energy, modality, technique, etc. as separate items, whereas we chose to consolidate this item as prescription (see #4 in Table 2). The use of clearcheck also enabled us to consolidate a number of items such as grid size, couch insertion, heterogeneity check, etc. into a single item. The one item that is on the TG275 list but not on ours is “Special Considerations for radiotherapy (e.g., pacemakers, ICDs, pumps, etc.).” In our department, there is a separate process and workflow to deal with implanted devices which the second checker does not have to deal with and hence it is not listed in our second checklist.

Our current study has several limitations; one is that the scoring system is subjective. We have tried to address this by discussing each failure mode and the consequent severity and the ability to detect each failure amongst the team members. In order to reduce the randomness of the “frequency of occurrence” scoring, all team members were shown the frequencies of incidents in the local database. The second limitation is the local database in itself. The accuracy of frequency is dependent on the ability of our department to capture all incidents in this database. The third limitation is the somewhat arbitrary choice of the cutoff score value (20) chosen for an item to be listed in the checklist. If the value is too large we may omit some important items and if it is too small then, there may be too many items on the checklist. We tried to mitigate this concern by adding another criteria, which is, any item with a severity score of 4 or greater is also placed on the checklist.

### 4.A Future work

One of the key recommendations of TG275 is to incorporate a formal physics review of critical data earlier in the treatment planning and not rely solely on the second check which is done at the end of planning. This will certainly be helpful in catching errors earlier on in the process and avoid a last minute replan or addressing of errors. We are assessing the feasibility of doing this in terms of man hours required, the work process, and the timeliness of getting this done without slowing down the planning process.

We plan to evaluate the effectiveness of the new checklist in error detection by comparing the error detection rate pre‐ and postimplementation of the new checklist as items that are not on the list may not be checked.

## 5 CONCLUSION

Failure Mode and Effects Analysis proved to be a valuable tool in identifying vulnerabilities within our workflow and processes in generating a treatment plan, subsequently leading to the creation of a new initial plan checklist that may be both more efficient and effective.

As radiation treatment technology advances, the changes in the clinical workflow is constant. Therefore, even after the initial second checklist is established, it is equally important to maintain a systematic periodic review of the second check process to ensure that the checklist is kept efficient and effective.^10^ It will also be beneficial to review the list, when new software, upgrades, programs, or technologies are introduced into the clinic.

## CONFLICT OF INTEREST

None.
